# Perceptually Salient Regions of the Modulation Power Spectrum for Musical Instrument Identification

**DOI:** 10.3389/fpsyg.2017.00587

**Published:** 2017-04-13

**Authors:** Etienne Thoret, Philippe Depalle, Stephen McAdams

**Affiliations:** Schulich School of Music, McGill UniversityMontreal, QC, Canada

**Keywords:** spectrotemporal modulation, musical timbre, Instrument identification, Modulation power spectrum, Bubble method

## Abstract

The ability of a listener to recognize sound sources, and in particular musical instruments from the sounds they produce, raises the question of determining the acoustical information used to achieve such a task. It is now well known that the shapes of the temporal and spectral envelopes are crucial to the recognition of a musical instrument. More recently, Modulation Power Spectra (MPS) have been shown to be a representation that potentially explains the perception of musical instrument sounds. Nevertheless, the question of which specific regions of this representation characterize a musical instrument is still open. An identification task was applied to two subsets of musical instruments: tuba, trombone, cello, saxophone, and clarinet on the one hand, and marimba, vibraphone, guitar, harp, and viola pizzicato on the other. The sounds were processed with filtered spectrotemporal modulations with 2D Gaussian windows. The most relevant regions of this representation for instrument identification were determined for each instrument and reveal the regions essential for their identification. The method used here is based on a “molecular approach,” the so-called bubbles method. Globally, the instruments were correctly identified and the lower values of spectrotemporal modulations are the most important regions of the MPS for recognizing instruments. Interestingly, instruments that were confused with each other led to non-overlapping regions and were confused when they were filtered in the most salient region of the other instrument. These results suggest that musical instrument timbres are characterized by specific spectrotemporal modulations, information which could contribute to music information retrieval tasks such as automatic source recognition.

## Introduction

Automatic musical instrument recognition is one of the more complex problems in musical informatics research. Work on how humans do this could provide important insights concerning how to get machines to do it, as well to improve automatic annotation algorithms, for example. Listeners' ability to recognize musical instruments has animated research for many years. From several points of view, either purely computational (Brown, 1999; Brown et al., 2001) or purely perceptual (McAdams, 1993, 2013), it has been shown that the acoustic signal encompasses many indices specific to each instrument, which contribute to their recognition. In order to understand what information is essential for algorithms or for perceptual recognition processes, mathematical representations of sound signals have been developed. In a discussion of the relation between Music Information Retrieval (MIR) issues and music cognition issues, Aucouturier and Bigand (2013) stressed the importance of investigating and developing biologically inspired representations to better understand what signal information is relevant in MIR tasks (see also Siedenburg et al., 2016), and reciprocally, how MIR algorithms may help to better understand the processing underpinning perceptual tasks.

The simplest representation of a sound is its waveform, which corresponds to the sound pressure recorded by a microphone or the vibration that moves the tympanic membrane. This first type of representation leads to timbre descriptors that are relevant either from a computational point of view or that have been shown to significantly contribute to perceptual dissimilarity judgments. For instance, attack time has been shown to be a strong perceptual cue to distinguish sustained and impulsively excited instruments (Iverson and Krumhansl, 1993; McAdams et al., 1995), and has also been shown to be a relevant feature for instrument classification (Saldanha and Corso, 1964). Nevertheless, this representation doesn't reveal many of aspects of a sound, in particular its spectral content. In order to reveal the evolution of the spectral content over time, spectrograms of sounds have been used for some time (Koenig et al., 1946). Interestingly, this representation can be related to the transformation of mechanical waves into neural signals achieved at the cochlear level. Many sound descriptors have been derived from this kind of representation. One of the most well-known is certainly the average spectral centroid over the duration of a sound, which has been shown to correlate well with perceptual dimensions (e.g., Grey and Gordon, 1978; McAdams et al., 1995; Giordano and McAdams, 2010; Hjortkjær and McAdams, 2016).

Many experiments using identification, discrimination or dissimilarity-rating tasks have investigated the specific influence of temporal and spectral cues on timbre perception. Hall and Beauchamp (2009), for example, have shown in identification and discrimination tasks that listeners are more sensitive to the spectral envelope of musical instrument sounds than to the temporal envelope, and they are more sensitive to spectral envelope shape than to the spectral centroid *per se*. In a meta-analysis of 23 datasets from 17 published studies, Giordano and McAdams (2010) showed that confusions in identification tasks are related to perceived similarity between the same instruments. These experiments have stressed that perceptual results can be explained to a certain extent by audio descriptors computed from spectral and spectrotemporal descriptors that are plausibly used by the auditory system to identify a sound source such as a musical instrument.

Recent studies have emphasized the interest of another kind of representation, the Modulation Power Spectrum (MPS) (Elliott and Theunissen, 2009; Elliott et al., 2013). Basically, the MPS corresponds to the two-dimensional Fourier transform of a spectrogram and can be seen as a representation characterizing its temporal and spectral periodicities. This representation highlights the temporal and spectral regularities of a spectrogram. For musical sounds with tremolo (regular amplitude modulation) for example, the MPS will be composed of a local maximum at the tremolo frequency. Similarly, if the musical sound is perfectly harmonic, the MPS will be composed of a local maximum in the spectral modulation dimension. Interestingly, as with the waveform or the spectrogram, this representation can be associated with a processing stage in the auditory system. Indeed, some neuron populations in primary auditory cortex seem to respond selectively to specific spectrotemporal modulations, at least in the ferret (Shamma, 2001). The prominent role of these spectrotemporal modulations in the perception and classification of musical timbre has been suggested recently (Patil et al., 2012; Elliott et al., 2013; Hemery and Aucouturier, 2015; Patil and Elhilali, 2015). In particular, Patil et al. (2012) have shown that this kind of representation can be used in the automatic classification of musical instruments, but it also correlates with perceptual dissimilarity ratings between instruments. Nevertheless, it remains unknown whether specific aspects of spectrotemporal modulations are relevant for the recognition of musical instruments. If some ranges of spectrotemporal modulation are more relevant than others to recognize and identify musical instruments, this would shed light on a possible strategy used by auditory processes to identify specific sound sources such as musical instruments. From a purely computational point of view, this approach would enable us to envisage new timbre descriptors related to musical instruments in addition to those derived from temporal and time-frequency representations (Peeters et al., 2011). Note that these potential timbre descriptors based on the MPS representation should also be linked to the timbre descriptors defined on time-frequency representations. As the spectral modulations are a kind of decomposition of the spectral envelope, MPS-based timbre descriptors should be linked to descriptors such as the formants, the spectral centroid, higher-order statistical moments or mel-frequency cepstral coefficients. For more detail concerning audio descriptors related to timbre perception, see Pachet and Aucouturier (2004), Peeters et al. (2011), and Elliott et al. (2013).

Here we tackle these questions for sustained (blown and bowed) instruments (tuba, trombone, saxophone, clarinet, cello) and instruments producing impulsive (plucked and struck) sounds (viola pizzicato, guitar, harp, vibraphone, marimba). We aimed to determine which region of the MPS leads to the identification of these musical instruments. Based on a filtering method proposed by Elliott and Theunissen (2009) and on a “molecular” approach, the so-called “bubbles” method, proposed by Gosselin and Schyns (2001), we set up an identification task in which listeners had to recognize processed versions of original sounds composed from a small region of their MPS. This allows us to determine the relevance of the location of each bubble, i.e., corresponding to a 2D Gaussian window, of the MPS in the recognition of musical instrument sounds and then, by combining the responses for bubble regions, to compute a global mask that highlights the most salient MPS regions for each instrument and for all instruments combined. This approach allows us to identify the most salient regions of the MPS for instrument identification, and moreover, if instruments are confused with each other, to determine which regions of the MPS lead to the specific confusions. The bubble method was initially developed to identify which part of a face is used by the visual system to determine gender and whether the face was expressive or not. Participants were asked to identify gender or categorize it as expressive or not from small parts of the face. A similar method has recently proved its efficacy in identifying which regions of the MPS are relevant for speech intelligibility (Venezia et al., 2016).

## The modulation power spectrum of musical sounds

**Figure 1:**
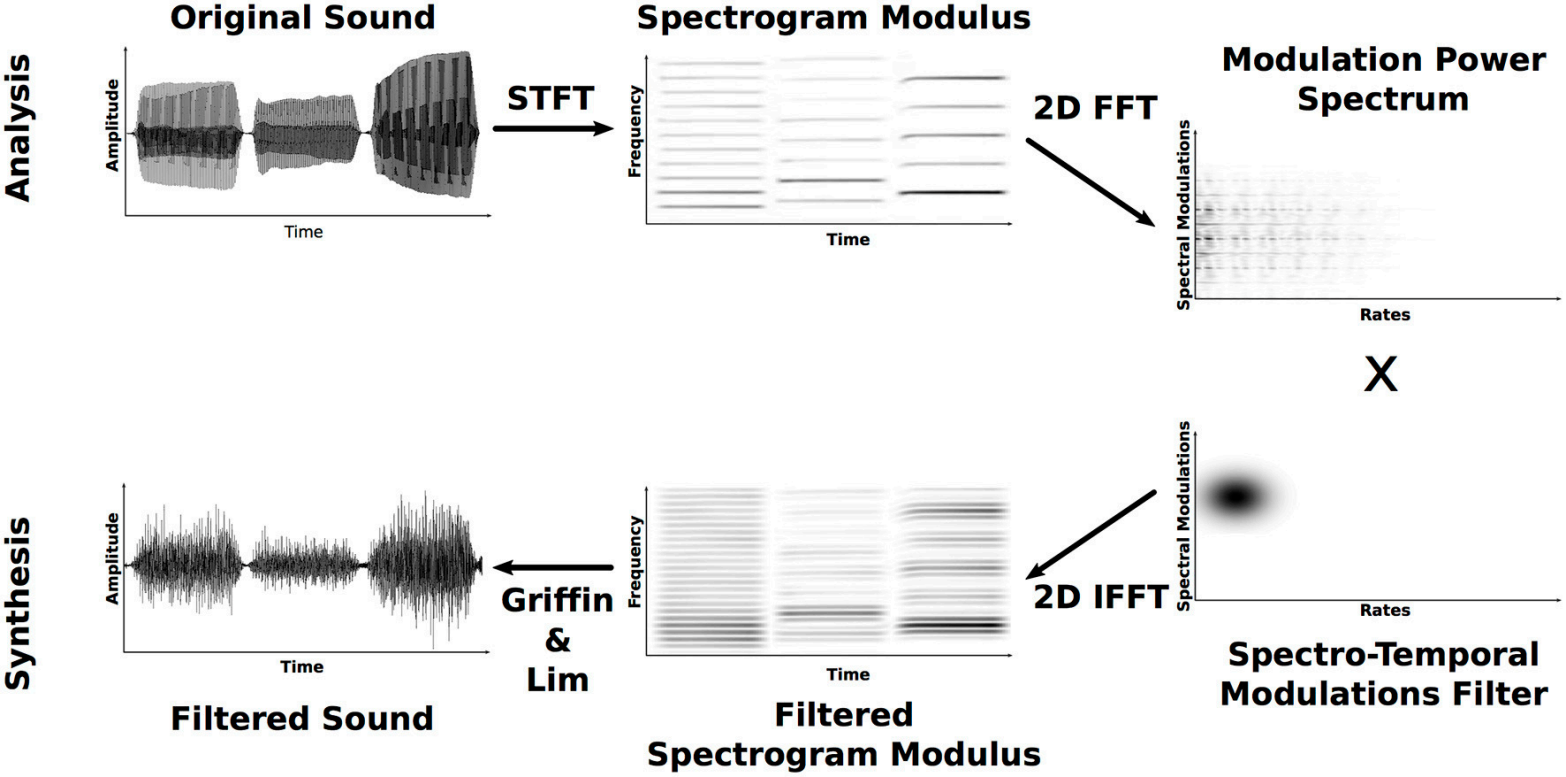
**Analysis–filtering–synthesis process**. Only the top-right quadrant of the MPS and of the filter are represented.

The MPS is defined here as the two-dimensional Fourier transform of the time-frequency representation (TFR) of a sound signal (Singh and Theunissen, 2003; Elliott and Theunissen, 2009). More specifically, the TFR *X*(*t, f*) itself is defined here as the amplitude of the Fourier transform obtained with a Gaussian window and is commonly known as the magnitude of the Short-Term Fourier Transform (STFT) or the Gabor Transform. The MPS is the amplitude of the successive Fourier transforms along the STFT temporal and frequency axes. This MPS representation is composed of two dimensions: temporal modulations (in Hz) and spectral modulations (in cycles/Hz), see Figure 1.

The resolution of the MPS, denoted *MPS*(*s, r*) with s and r being spectral and temporal modulations, respectively, is constrained by the resolution of the time-frequency representation *X*(*t, f*), mainly characterized by the effective sizes of the temporal Gaussian windows and the overlap between two successive windows. They indeed define the upper and lower boundaries of the spectral and temporal modulations axes. Constrained by the uncertainty principle $\sigma_{t}\geq \frac{1}{2\pi \sigma_{f}}$ where σ_*t*_ and σ_*f*_ correspond to the uncertainties along the temporal and spectral modulation dimensions, respectively, we here choose σ_*t*_ = 11.61 ms and σ_*f*_ = 21.53 Hz leading to upper boundaries of 43 Hz and 23.22 cycles/Hz which correspond to values relevant for the auditory perception of sounds such as speech (Elliott and Theunissen, 2009).

## Experiments 1 and 2

### Materials and methods

#### Participants

Thirty-one participants (12 females) with ages between 19 and 45 (*M* = 24.4, *SD* = 5.7) took part in the first experiment and 32 participants (14 females) with ages between 18 and 45 (*M* = 24.2, *SD* = 5.7) took part in the second experiment. All participants were musicians who had completed at least second-year university-level musical training in performance, composition or theory. Seventeen of the participants took part in both experiments (5 females). Participants provided informed consent, had normal hearing, and were compensated for their time.

#### Stimuli

The stimuli were five arpeggios generated from samples of the Vienna Symphonic Library. In the first experiment, five sustained instruments (trombone, tuba, saxophone, cello, and clarinet) playing three musical pitches: F#3 (with a fundamental frequency of 185.0 Hz), C4 (261.6 Hz), and F#4 (370.0 Hz) were chosen. This range of pitches doesn't involve large variations of timbre across the three different notes. In the second experiment, five impulsive instruments were chosen (vibraphone, marimba, harp, guitar, viola pizzicato) playing the same pitches. Based on other work in the lab (McAdams et al., 2016), we chose to separate sustained instruments from impulsive instruments as it would have been too obvious to distinguish them in an identification task. For each instrument, the three notes were equalized in loudness in a preliminary experiment. Their durations were all cut to 0.5 s with a 50-ms raised cosine fade-out amplitude envelope to avoid discrimination based on duration. The attack was preserved. Finally, arpeggios were generated by concatenating the three notes from the lowest to the highest.

In order to determine which regions of the MPS lead to the identification of musical instruments, we employed a technique for filtering instrumental sounds in the spectrotemporal modulation domain (see Figure 1). With this technique, a sound is processed by keeping only a small region of its MPS, this filtered version is reconstructed, and then whether the information that remains is relevant for the identification of the initial instrument is evaluated with listener testing. Hence, the MPS is first multiplied by a “bubble,” a two-dimensional Gaussian MPS-filter frequency response *G*_(_μ__*s*_, σ_*s*_), (μ_*r*_, σ_*r*_)_(*s, r*) where μ_*s*_, μ_*r*_ and σ_*s*_, σ_*r*_ are the means and standard deviations in the scale and rate dimensions, respectively:

(1) $$\begin{array}{l} \begin{array}{ccc} G_{\left(\mu_{s},\sigma_{s}),(\mu_{r},\sigma_{r}\right)}(s,r) & = & \exp\left(-\frac{1}{2}{\left(\frac{s-\mu_{s}}{\sigma_{s}}\right)}^{2}\right) \end{array}\\ \;\exp\left(-\frac{1}{2}{\left(\frac{r-\mu_{r}}{\sigma_{r}}\right)}^{2}\right) \end{array}$$

It must be noted that the MPS and the filter *G* are composed of four quadrants with positive and negative spectral and temporal modulations. For the sake of simplicity and as the filter is perfectly symmetric in amplitude and phase in the spectral and temporal modulation dimensions, only positive values are presented in what follows. The MPS-filtered TFR *Y*(*t, f*) can then be easily reconstructed by a 2D inverse Fourier transform of the processed MPS: *MPS*(*s, r*)·*G*_(_μ__*s*_, σ_*s*_), (μ_*r*_, σ_*r*_)_(*s, r*). Note that *Y*(*t, f*) is magnitude only, lacks the phase, and thus does not allow for perfect reconstruction of the waveform directly from standard reconstruction technique such as the overlap-add method (OLA; Rabiner and Schafer, 1978). Therefore, we instead used Griffin and Lim's (1984) algorithm in a MATLAB implementation provided by Slaney (1994) in order to iteratively build a signal, the STFT magnitude of which is as close as possible to the *Y*(*t, f*) in a quadratic sense. Twenty-five iterations lead to a correct reconstruction of the waveform for an acceptable computation time. Figure 1 summarizes the whole analysis-filtering-synthesis process. Practically speaking, the quality of the reconstruction is evaluated by computing the averaged relative log-error ratio ϵ in percent between the desired spectrogram *Y*(*t, f*) and the STFT magnitude of the reconstructed waveform *Y*_*b*_(*t, f*):

(2) $$\epsilon =100\frac{1}{N_{f}N_{t}}{\sum^{}}_{t_{i=1}}^{N_{t}}{\sum^{}}_{f_{i=1}}^{N_{f}}\left|\frac{\log(Y(t_{i},f_{i}))-\text{log}(Y_{b}(t_{i},f_{i}))}{\text{log}(Y(t_{i},f_{i}))}\right|$$

where *N*_*f*_ and *N*_*t*_ are the numbers of frequency and time bins, respectively.

The stimulus files were normalized at −3 dB relative to 16-bit amplitude resolution. In the first experiment, the peak level of the stimuli ranged from 58 to 71 dB SPL (A-weighted). In the second experiment, the peak level of the stimuli ranged from 63 to 70 dB SPL (A-weighted). Stimuli were classically sampled at 44,100 Hz with 16-bit resolution.

#### Apparatus

Both experiments took place in an IAC model 120act-3 double-walled audiometric booth (IAC Acoustics, Bronx, NY). Stimuli were presented over Sennheiser HD280Pro headphones (Sennheiser Electronics GmbH, Wedemark, Germany) using a Macintosh computer (Apple Computer, Inc., Cupertino, CA) with digital-to-analog conversion on a Grace Design m904 monitor system (Grace Digital Audio, San Diego, CA). The experimental interface was programmed in the Max7 audio software environment (Cycling '74, San Francisco, CA) and data collection was programmed in Matlab (The Mathworks, Inc., Natick, MA) interacting via the User Data Protocol (*udp*).

#### Procedure

**Figure 2:**
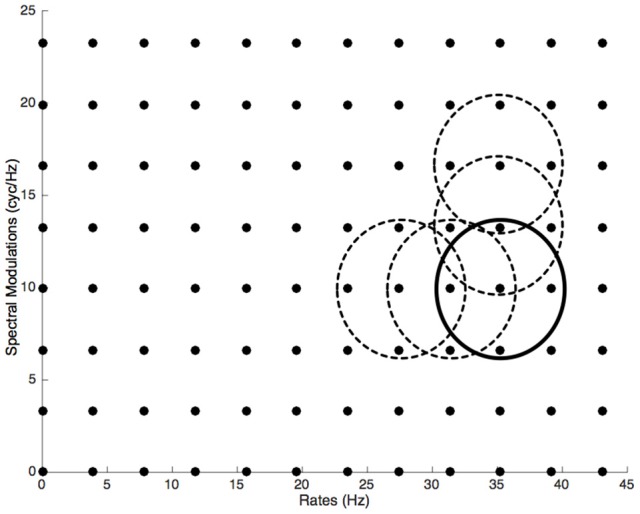
**Sampling of the Modulation Power Spectrum by 96 Gaussian filters in the scale-rate plane**. The dots show the center value and the circles the standard deviation of the 2D Gaussian distribution.

Participants first completed a standard pure-tone audiogram to ensure normal hearing with hearing thresholds of 20 dB HL or better at octave-spaced frequencies in the range of 250–8,000 Hz (Martin and Champlin, 2000; ISO 389–8, 2004). The task was 5-Alternative Forced Choice (5-AFC). In each trial, the participants were asked to recognize the instrument that played the arpeggios among the five instruments. They were asked to answer as quickly as possible after hearing the sounds in order that they answer the most intuitively when the sounds were degraded by the filtering process. The experiment began with a training session of 15 trials (5 instruments × 3 repetitions) during which the participants performed the task with the original, unprocessed sounds. After having completed the training session, the participants began the main experiment, which was composed of 480 trials (5 instruments × 96 filters). For each instrument, the MPS was filtered with 96 Gaussian filters *G*_(_μ__*s*_, σ_*s*_), (μ_*r*_, σ_*r*_)_ with the following standard deviations: σ_*r*_ = 5 *Hz* and σ_*s*_ = 4 *cycles*/*Hz* overlapping by 75% along each dimension (12 rates and 8 spectral modulations, see Figure 2). These standard deviations were determined by empirical tests in order to provide a good trade-off between accurate sampling and a reasonable number of filters for sampling the MPS. The averaged log-error ratio (cf. Equation 2) for the 480 sounds equaled 10.25%. Hence in each trial, one of the five instrument arpeggios was processed with one filter, and the participant had to recognize the original instrument. The order of presentation of the 480 trials was randomized for each participant.

#### Data analysis

For all participants and for all five instruments, a confusion matrix was computed and association scores were tested against chance level with a one-tailed *t*-test. The *p*-values were adjusted with Bonferroni corrections for multiple testing. The subsequent data analysis was inspired by the so-called “bubbles” method proposed by Gosselin and Schyns (2001). In each trial, if the sound was properly associated with the instrument, the MPS filter was added to a CorrectMask matrix. Across all trials, each MPS filter was added to a TotalMask matrix. For each participant, a ProportionMask was derived by dividing CorrectMask by TotalMask. If no region had any special perceptual significance for recognition, ProportionMask would be homogeneous. To the contrary, if some regions were more important for recognition, they would have higher values than the other regions of the ProportionMask. Note that our method differs from that of Gosselin and Schyns (2001), which was initially used to determine the most salient parts of a face for gender and expressivity recognition. Although they used an adaptive method that adjusted the number of bubbles to converge on 75% correct recognition, here we only used single bubbles in order to determine their independent contribution to instrument identification. Given that MPS filters overlap each other, the resulting ProportionMasks represent the relative importance of each region of the MPS to the identification of that instrument. In order to determine which regions are the most relevant for the identification of each instrument, a one-tailed *t*-test between ProportionMask values and the averaged value of the ProportionMask (α = 0.05) was applied for each instrument and across participants to compute a SalienceMask. Hence, the *p*-values of these tests were here used as a measure of the relevance of each spectrotemporal modulation value: the smaller the *p*-values, the more salient the spectrotemporal modulation. The statistical significance of each spectrotemporal modulation was also determined and corresponds to the DiagnosticMask of Gosselin and Schyns (2001). Here, we considered that a bin of the SalienceMask is significant when the *p*-value is lower than 0.05. The DiagnosticMask is a binary mask set to 1 or 0 when the SalienceMask is significant or not, respectively. The description of all of the masks described previously is summarized in Table 1.

**Table 1:** **Summary of the different Masks computed for the analysis of the salient regions of the MPS for each instrument**.

**Mask**	**Description**
CorrectMask	For one instrument, sum of the filters leading to correct identification.
TotalMask	Sum of all the filters.
ProportionMask	Ratio between the CorrectMask and the TotalMask.
SalienceMask	For each instrument, the *p*-value of a single-sample *t*-test against chance performance (0.2) of each bin of the CorrectMask.
ConfusionMask	Ratio between the sum of the filters leading to a wrong association of instrument A with instrument B and the sum of all filters.
DiagnosticMask	Binary mask associated with a SalienceMask or a ConfusionMask. For each bin, it equals 1 if the SalienceMask of ConfusionMask's bin is significant, i.e., *p* < 0.05, and equals 0 otherwise.

In order to reveal the most salient spectrotemporal modulation regions, we first computed the SalienceMask for all instruments, and then for each instrument separately. In addition, when one instrument is significantly confused with another one, the same analysis is performed to generate a ConfusionMask by substituting the correctly associated mask in the CorrectMask with those from the instrument with which it has been confused. This mask reveals the spectrotemporal regions in which one instrument is incorrectly identified as another.

### Results

#### Confusion matrices

Tables 2, 3 present the averaged confusion matrices across participants from the two experiments. All instruments were recognized above chance in both experiments [*p* < 0.001–Trombone: *t*_(30)_ = 12.84, *d* = 2.31, Clarinet: *t*_(30)_ = 16.28, *d* = 2.92, Tuba: *t*_(30)_ = 12.31, *d* = 2.21, Cello: *t*_(30)_ = 13.84, *d* = 2.48, Saxophone: *t*_(30)_ = 9.82, *d* = 1.76 for Experiment 1, and *p* < 0.001—Viola Pizzicato: *t*_(31)_ = 15.30, *d* = 2.70, Guitar: *t*_(31)_ = 8.02, *d* = 1.41, Harp: *t*_(31)_ = 11.49, *d* = 2.03, Marimba: *t*_(31)_ = 13.02, *d* = 2.30, Vibraphone: *t*_(31)_ = 10.57, *d* = 1.86 for Experiment 2]. In addition, in Experiment 1, tuba, cello and saxophone were significantly confused with trombone [*t*_(30)_ = 5.91, *p* < 0.001, *d* = 1.06], saxophone [*t*_(30)_ = 1.75, *p* < 0.05, *d* = 0.31] and cello [*t*_(30)_ = 3.84, *p* < 0.01, *d* = 0.69], respectively. In the second experiment, the guitar, harp, marimba and vibraphone were significantly confused with harp [*t*_(31)_ = 4.32, *p* < 0.001, *d* = 0.76], guitar [*t*_(31)_ = 3.69, *p* < 0.001, *d* = 0.65], vibraphone [*t*_(31)_ = 2.59, *p* < 0.01, *d* = 0.45] and marimba [*t*_(31)_ = 2.35, *p* < 0.05, *d* = 0.41], respectively.

**Table 2:** **Confusion matrix in percent response averaged across participants for experiment 1 (sustained sounds)**.

	**Trombone**	**Clarinet**	**Tuba**	**Cello**	**Saxophone**
Trombone	61^***^	2.6	20.6	3.5	12.3
Clarinet	3.6	69.9^***^	7	9.6	9.9
Tuba	34.8^***^	2.9	54.5^***^	3.3	4.5
Cello	4.1	7.3	5.7	59.6^***^	23.3^*^
Saxophone	5	7.6	5.5	30.9^**^	51^***^

*Association rates significantly above chance are shown in bold*.

*** p < 0.001;

** p < 0.01;

* *p < 0.05*.

**Table 3:** **Confusion matrix in percent response averaged across participants for experiment 2 (impulsive sounds)**.

	**Viola Pizz**.	**Guitar**	**Harp**	**Marimba**	**Vibraphone**
Viola Pizz.	69.8^***^	9.9	12.1	5.3	2.9
Guitar	9.1	45.2^***^	30.1^***^	7	8.6
Harp	16.6	27.5^***^	42.9^***^	7.3	5.7
Marimba	3	4.5	4	61.9^***^	26.5^**^
Vibraphone	0.6	1.6	1.1	30.1^*^	66.6^***^

*Association rates significantly above chance are shown in bold*.

*** p < 0.001;

** p < 0.01;

* *p < 0.05*.

## Perceptually relevant spectrotemporal modulations

**Figure 3:**
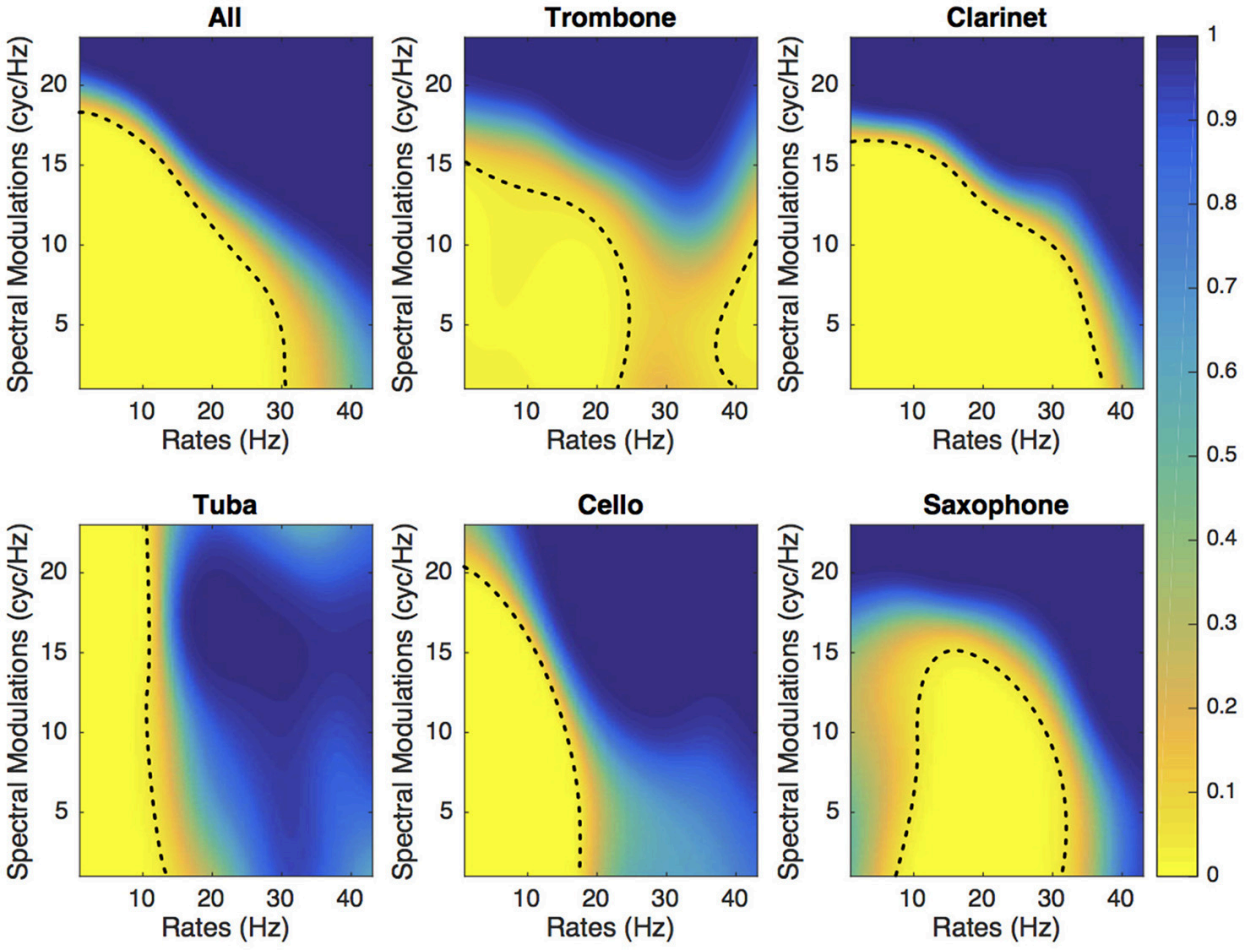
**Experiment 1**. SalienceMask of the MPS for the five sustained instruments and all instruments combined. The dashed line represents the threshold at *p* = 0.05.

**Figure 4:**
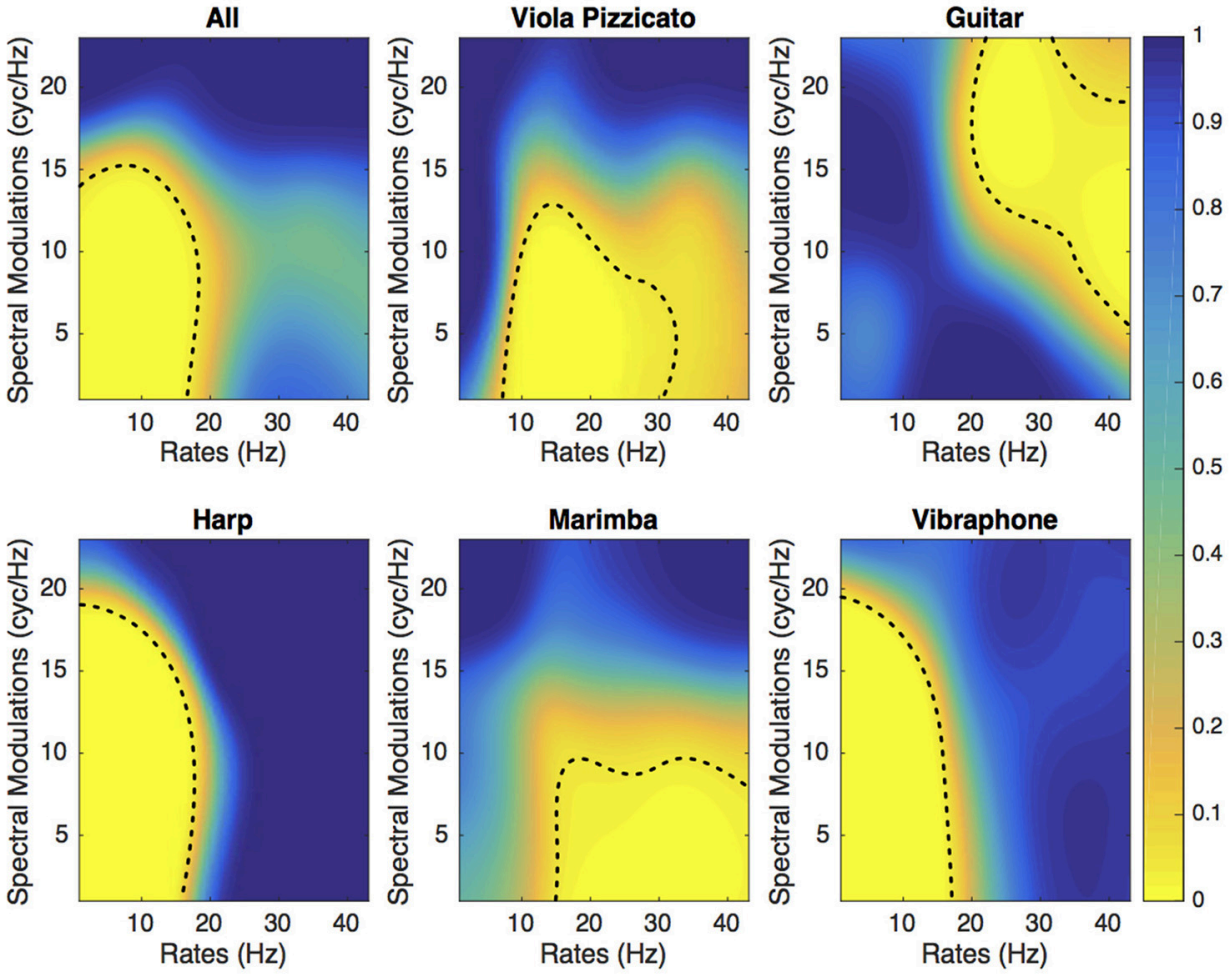
**Experiment 2**. SalienceMask of the MPS of the five sustained instruments and all instruments combined. The dashed lines represent the thresholds at *p* = 0.05.

Figures 3, 4 present the SalienceMask for all instruments combined and for each instrument separately for Experiments 1 and 2. The yellowest regions of each plot are the most salient regions of the MPS. The *p*-values of the ProportionMasks are displayed. Concerning the sustained sounds and for all instruments combined (upper left plot of Figure 3), the most salient spectrotemporal modulations ranged from 0 to 30 Hz and from 0 to 18 cyc/Hz. The trombone, the clarinet and the cello also have their most relevant regions for low spectral and temporal modulations (Figure 3). The saxophone has its most salient region for temporal modulations comprised between 10 and 30 Hz. Concerning the tuba, the whole range of spectral modulations is relevant for its identification. For impulsive sounds and all instruments combined (upper left of Figure 4), the most salient spectrotemporal modulations ranged from 0 to 18 Hz and from 0 to 15 cyc/Hz. The harp and the vibraphone also have their most relevant regions for low spectral and temporal modulation. The viola pizzicato has its most salient MPS regions comprised between 10 and 30 Hz and 0 and 15 cyc/Hz. The marimba has its most salient regions for high rates (>15 Hz). The guitar has its most salient regions for high rates (>20 Hz) and high spectral modulations (>5 cyc/Hz). It is interesting to note that in both experiments, the most relevant spectrotemporal modulations for all instruments combined are centered on the same region, i.e., low spectral and temporal modulations.

**Figure 5:**
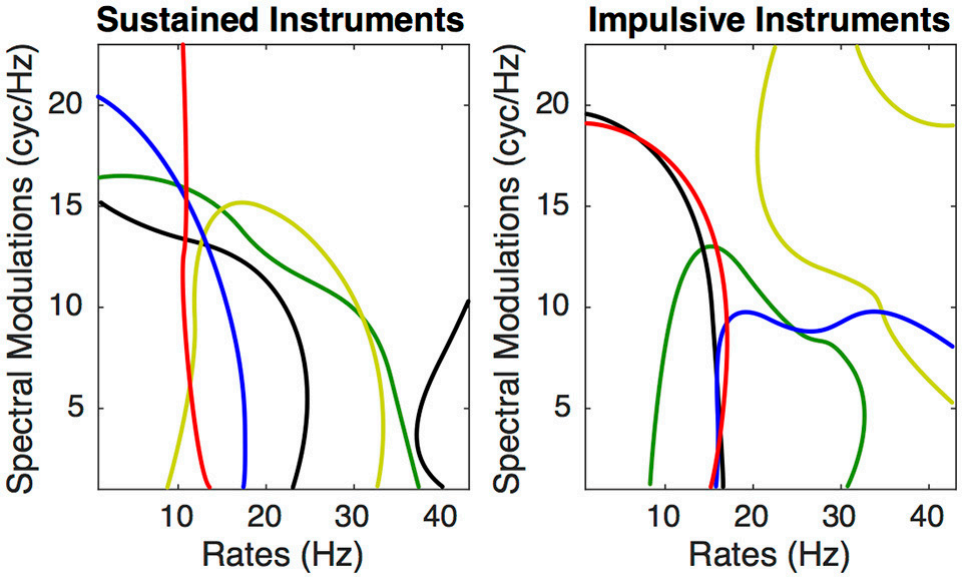
**DiagnosticMasks of the MPS of the five sustained instruments. Left:** Tuba red, Clarinet green, Saxophone yellow, Cello blue, Trombone black. **Right**: Viola Pizzicato green, Guitar yellow, Harp red, Marimba green, Vibraphone black.

If we consider the DiagnosticMask (see Figure 5), the most salient regions of the plane for all sustained instruments combined and all impulsive instruments combined represents 38 and 22.9%, respectively. If we consider each instrument separately, the sustaining instrument that provides the largest salient area is the clarinet (45.5% of the MPS plane) followed by saxophone (38.9%), trombone (33.4%), cello (28.3%), and tuba (25.3%). The five impulsively excited instruments have salient areas of similar size, 27.4% for viola pizzicato, 29% for guitar, 24.7% for harp, 27.1% for marimba and 24.6% for vibraphone.

**Figure 6:**
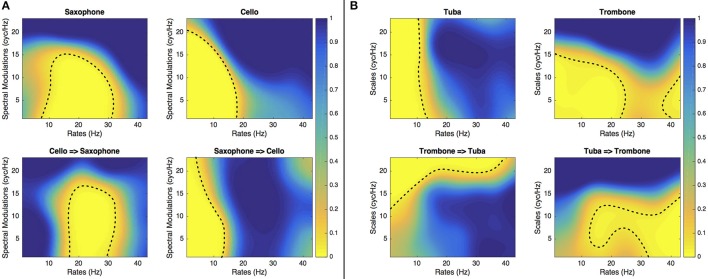
**Experiment 1**. *ConfusionMasks* of the cello/saxophone **(A)** and trombone/tuba **(B)**. The dashed lines represent the thresholds at *p* = 0.05. The upper panels show the instrument identified as itself and the lower panels show the instrument identified as another instrument.

**Figure 7:**
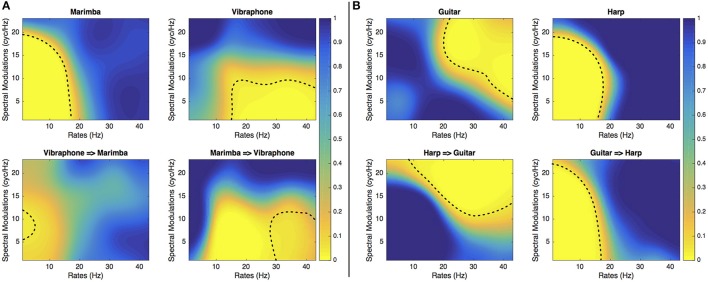
**Experiment 2**. *ConfusionMasks* of the marimba/vibraphone **(A)** and guitar/harp **(B)**. The dashed lines represent the thresholds at *p* = 0.05. The upper panels show the instrument identified as itself and the lower panels show the instrument wrongly identified as another instrument.

Interestingly, for instruments that were confused, the ConfusionMasks presented in Figures 6, 7 confirm that the salient regions of the SalienceMask lead to confusion when an instrument's MPS is filtered with spectrotemporal modulations in the most salient areas of the other instrument. For instance, the area leading to identifications of the cello stimulus as a saxophone corresponds to the most salient area of the saxophone and vice versa. The same phenomenon is observed for the marimba/vibraphone and harp/guitar pairs (see Figure 7) and to a certain extent for the trombone and the tuba (see Figure 6). These results confirm that these spectrotemporal areas are specific to the timbre of the confused instruments.

## Discussion

In this paper we sought to determine the most salient regions of the MPS for the identification of musical instruments producing either sustained or impulsive sounds. Based on the “bubbles” method developed by Gosselin and Schyns (2001), we have shown that globally the most salient spectrotemporal modulations are centered on low rates and low spectral modulations. Interestingly, when two instruments are confused, the spectrotemporal modulations enabling their discrimination do not overlap, suggesting that these regions are specific to these instruments. Moreover, note that confusions appear when the original sounds are filtered in the most salient regions of the instrument with which they are confused, reinforcing the idea that they are specific to the timbre of these instruments. Also, specific regions of the MPS other than the low spectral and temporal modulations are specific to some instruments, e.g., for the guitar. This does not concur with the general finding that globally low rates and low spectral modulations are relevant and suggests that when instruments were confused, listeners were focusing on a specific region of the MPS.

From a perceptual point of view, the fact that different regions of the MPS are more or less significant for the identification of different instruments suggests that these regions are specific to the timbre of these instruments. Counterintuitively, we could have thought that instruments sharing the same relevant region would be confused. However, the SalienceMasks reveal the region that allows for identification within the context of the sound set being tested. Two instruments can therefore have close SalienceMasks and even provide good recognition, suggesting that the SalienceMasks cannot be used as a measure of similarity between instruments. Conversely, when two instruments are confused, the fact that their salient spectrotemporal modulations don't overlap, and, even more, that their ConfusionMask falls within the region of the SalienceMasks of the other instrument, reinforces the idea that these two non-overlapping regions are specific to these instruments in this context. For example, according to these results, we can conclude that the SalienceMask of the saxophone corresponds to specific timbral properties of this instrument in comparison with those of the cello timbre with which it has been confused. Nevertheless, we suspect that if the cello had been removed from the instrument subset, the SalienceMasks of the saxophone would have been different. The same expectation would hold for the trombone/tuba, guitar/harp and marimba/vibraphone pairs as well.

In order to fully validate that specific MPS regions are characteristic of some instruments, additional experimentation is needed. In particular, an identification experiment with the original sounds filtered by their SalienceMasks would evaluate whether it removes the confusions between the different instruments. From a cortical point of view, we may expect that this ability to focus on different regions of the MPS is possible due to the plasticity of the neurons in primary auditory cortex. Several studies have indeed revealed that neurons of this cortical network can reshape their sensitivity to different spectrotemporal modulations according to the needs of the tasks (Fritz et al., 2003; David et al., 2012; Slee and David, 2015). It is therefore possible in the context of each instrument subset that our cognitive processes can focus on different regions of the MPS in order to discriminate similar instrument sounds within a given stimulus context.

These results can also be considered in the light of the recent study of Isnard et al. (2016) who showed that severely impoverished sounds in the time-frequency domain—music, speech or environmental sounds—can still be recognized. In the same way, Suied et al. (2013) determined a perceptually sparse representation of speech sounds in the spectrotemporal modulation domain in order to determine the minimum acoustic information necessary to convey emotions in speech sounds. In line with this work, we have shown here that musical instrument sounds impoverished in the spectrotemporal modulation domain can still be recognized.

From a more general perspective, these two experiments are a first step toward determining new acoustic descriptors relevant to the perception of musical timbre. Even if the MPS appears to be less intuitive than the time-frequency representation, it must be noted that it is an ingenious way to describe the spectrum of a sound as it is invariant according to several transformations in the time-frequency domain. Here, we considered a spectrogram with a linear frequency scale for which the MPS is invariant by translation in the time-frequency domain. Hence we may expect to determine acoustical invariants that characterize musical instruments categories (McAdams, 1993) from these representations.

## Conclusion

The results of this study shed light on the most relevant regions of the MPS for the identification of musical instrument timbre. From a perceptual point of view, this research provides a ground from which to investigate whether the MPS regions determined here could be used to determine new timbre descriptors and/or serve as a sound representation for automatic recognition algorithms. Moreover, comparison with other approaches to timbre such as multidimensional scaling might be an interesting perspective of this work, although Elliott et al. (2013) found fairly similar predictive power for MPS representations and combinations of unidimensional audio descriptors. Future research will focus on how this new approach is linked to the other conceptions of timbre. In particular, we can expect to link temporal modulations to the relevant aspects of the temporal envelope (e.g., the attack time) and similarly with spectral modulation and spectral envelope properties (e.g., formant and pitch). As the stimuli were composed of arpeggios, no specific analysis has been done on how filtering in the MPS domain might impact properties such as attack time for each note. It is for instance plausible that the filtering in the temporal modulation dimension may have impacted rise times. Moreover, other parameters such as the loudness of the filtered stimuli may have influenced the identification scores and could also be investigated in further experiments, although it isn't clear how to “control” for this factor given that the filtered signals in different regions of the MPS have differing amounts of energy. Finally, it might be of interest to compare the relevance of the MPS representation with other spectrotemporal modulation representations such as those used by Patil et al. (2012) or Andén et al. (2015) inspired by the plausible two-dimensional wavelet achieved at the level of the primary auditory cortex by spectrotemporal receptive fields (Shamma, 2001).

## Ethics statement

The protocol of this study was certified for ethics compliance by the McGill Research Ethics Board II with written consent from all subjects in accordance with the Declaration of Helsinki.

## Author contributions

ET, PD, and SM conceived and designed the experiments. ET performed the experiments. ET, PD, and SM analyzed the data. ET, PD, and SM wrote the paper.

## Funding

This work was supported by grants from the Natural Sciences and Engineering Research Council of Canada awarded to SM (RGPIN-2015-05208, RGPAS-478121-15) and to PD (RGPIN-262808-2012) as well as a Canada Research Chair awarded to SM.

### Conflict of interest statement

The authors declare that the research was conducted in the absence of any commercial or financial relationships that could be construed as a potential conflict of interest.
